# Modulating Neuroinflammation to Treat Neuropsychiatric Disorders

**DOI:** 10.1155/2017/5071786

**Published:** 2017-10-18

**Authors:** Franziska A. Radtke, Gareth Chapman, Jeremy Hall, Yasir A. Syed

**Affiliations:** ^1^Neuroscience and Mental Health Research Institute and School of Biosciences, Cardiff University, Hadyn Ellis Building, Maindy Road, Cardiff CF24 4HQ, UK; ^2^MRC Centre for Neuropsychiatric Genetics and Genomics, Cardiff University, Hadyn Ellis Building, Maindy Road, Cardiff CF24 4HQ, UK

## Abstract

Neuroinflammation is recognised as one of the potential mechanisms mediating the onset of a broad range of psychiatric disorders and may contribute to nonresponsiveness to current therapies. Both preclinical and clinical studies have indicated that aberrant inflammatory responses can result in altered behavioral responses and cognitive deficits. In this review, we discuss the role of inflammation in the pathogenesis of neuropsychiatric disorders and ask the question if certain genetic copy-number variants (CNVs) associated with psychiatric disorders might play a role in modulating inflammation. Furthermore, we detail some of the potential treatment strategies for psychiatric disorders that may operate by altering inflammatory responses.

## 1. Introduction

Neuropsychiatric disorders including devastating diseases such as schizophrenia, major depressive disorder, and bipolar disorder are generally considered to have a multifactorial pathophysiology including both genetic and environmental factors [1]. Neuroinflammation could be one of the potential mechanisms contributing to pathogenesis, anchored in the interplay of environmental factors such as hypoxia or infections and genetic susceptibility of the immune system.

In fact, increasing amount of evidence suggests that inflammatory processes have an important role in the pathophysiology of psychiatric disorders. The significantly higher level of different inflammatory markers such as cytokines, chemokines, and chemokine receptors in patients suffering from various forms of psychiatric disorder has laid molecular foundation for the important role of inflammation in the pathogenesis of neuropsychiatric disorder [2–6]. Furthermore, there is increasing evidence from genetic studies that these altered immune processes may play a primary role in the development of neuropsychiatric disorders, rather than simply being a consequence of associated brain pathology. Assuming inflammation plays a key role in the pathogenesis of psychiatric disorders, anti-inflammatory treatments may play a critical role in the treatment of these disorders.

To what extent and in which case has this anti-inflammatory therapy already been applied successfully is far from clear. The efficacy of prescribing anti-inflammatory drugs to treat depression and other psychiatric diseases, either alone or in conjunction with traditional medication, remains to be elucidated. In this review, we will try to summarize the answers to these questions and sum up treatment recommendations where available.

## 2. Relationship between Inflammation and Mental Illness

**Figure 1:**
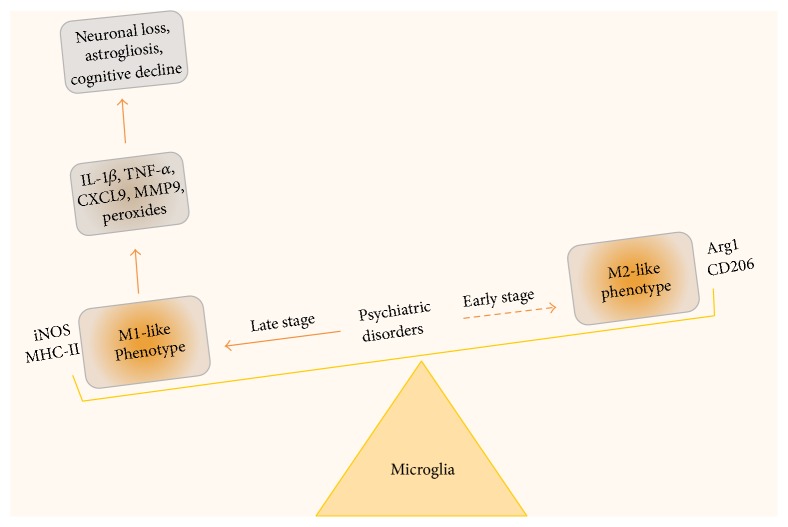
*Potential mechanism of microglia activation in psychiatric disorders.* Neuroinflammation is one of the key components of the pathogenic mechanisms underlying several psychiatric disorders and is often associated with microglial activation/dysfunction. Accumulating evidence indicate that M1-like microglia (proinflammatory) are significantly increased in comparison to ramified microglia (resting) and elongated M2-like microglia (anti-inflammatory) phenotypes in disease states. The levels of M1-like microglia in brain predominate and potentially can be associated with the severity of the disease, suggesting an imbalance in M1/M2 phenotype. M1-like microglia are characterized by the expression of MHC class II antigens and by the production of proinflammatory cytokines and nitric oxide synthase (iNOS). Continued production of proinflammatory cytokines can lead to neuronal damage, astrogliosis, plasticity, and cognitive decline. Peripherally derived macrophages and monocytes also participate in the inflammatory response. It is likely that during early stage of disease onset microglia can have phenotypic switch to an alternative state knows as M2-like phenotype, which are characterized by presence of surface markers like Arginase 1 and mannose receptor CD206, leading to resolution of inflammatory response by secretion of anti-inflammatory cytokines. The efficacy of the anti-inflammatory drug targeting M1/M2 balance will significantly depend on therapeutic time window and severity of symptoms associated with the diseases.

Recent studies on preclinical, genetics, and bioinformatics data have shown the activation of immune system molecules and pathways that can contribute to pathogenesis of psychiatric disorders [7]. Several lines of the evidence that support a role for inflammation as a contributing factor in psychiatric disorders include the following.

(I) It has been established that cytokines that are found typically during an ongoing inflammatory process are found to be elevated in blood samples of patients with various types of psychiatric disorders. This, depending on the study, includes both generally considered proinflammatory (i.e., interleukin- (IL-) 1-3, IL-5-9, IL-11-18, interferons (IFN), tumor necrosis factor (TNF), and chemokines) as well as anti-inflammatory (i.e., IL-4, IL-10, IL-11, and IL-13) cytokines and complement factors. Though the activation cascade of this elevated cytokine production is not yet understood, the findings may point to a significant role of peripheral inflammatory processes in psychiatric conditions. Following examination of blood samples from patients with schizophrenia [8, 9], depression [10, 11], anxiety [12], bipolar disorder [13–15], obsessive-compulsive disorder (OCD) [16, 17], posttraumatic stress disorder (PTSD) [18, 19], and autism spectrum disorder [20, 21] significantly elevated levels of all major kinds of cytokines were detected. This also included soluble interleukin receptors, interleukin antagonists, TNF, soluble TNF receptor IFN-*γ*, chemokines, and matrix metalloproteinases (MMP) (see Figure 1 and Table 1). The levels of some of these cytokines have also been correlated in some studies to the severity of disease symptoms [12, 17]. Work on the Whitehall II cohort shows that individuals with increased IL-6 levels over a prolonged period of time (one, two, or three measurements over a 5-year period) have an elevated subsequent 10-year risk for development of cognitive symptoms of depression [22]. There are multiple studies showing elevated high sensitivity- (hs-) C-reactive protein (CRP) levels in blood samples from patients with psychiatric disorders (schizophrenia, depression, PTSD, anxiety, and autism). Elevated levels of proinflammatory cytokines like IL-6 and IL-1*β* can lead to the production of this acute phase protein in the liver [14, 23–28]. Importantly, a meta-analysis of 8 longitudinal studies showed that increased CRP levels are significantly associated with the risk for later development of depressive symptoms. This result was independent of age and a range of other risk factors of depression [24]. Interestingly, higher levels of CRP and IL-6 during childhood (age of nine years) were shown to be a predictor of higher risk of depression and psychosis in later life [29]. Increased CRP was even suggested as a diagnostic tool of autism spectrum disorder due to a positive correlation with symptom severity [27].

It is difficult to examine cytokine and acute phase protein levels in the brain directly, hence findings from the peripheral blood therefore have to be interpreted with caution. This is why it is worth looking, among other aspects, into gene expression when reviewing results from postmortem samples of the brain.

(II) In schizophrenia, transcriptome analysis of brain tissue from autopsy of schizophrenic patients showed an increased expression of IFITM2, IFITM3, SERPINA3, and GBP1 was found. These genes are directly regulated by TNF-*α*, IFN-*α*, and IFN-*γ*, suggesting a proinflammatory state [30]. A recent study examining gene expression of the dorsolateral prefrontal cortex from patients with mood disorders demonstrate an elevated expression of inflammatory agents (IL-1*α*, IL-2, IL-3, IL-5, IL-8, IL-9, IL-10, IL-12A, IL-13, IL-15, IL-18, IFN-*γ*, and lymphotoxin-*α*) compared to control subjects [31]. Therefore, these results suggest that the findings seen using blood samples may be recapitulated in gene expression of key inflammatory agents in the brain, albeit in postmortem samples. A systematic review of 119 publications on postmortem tissue investigating further evidence of inflammation in schizophrenia concluded that there is high variability between the results found in different studies. For astrocyte or microglia markers no consistent increase or decrease was found across different studies. These investigations varied in patient age, diagnosis, and brain region examined. Out of 33 studies, 6 showed an increase in the astrocyte marker, glia fibrillary acidic protein (GFAP), while 6 showed a decrease. An increase in microglial markers (CD68, human leukocyte antigens (HLA), MHC antigens, CD11b, calprotectin, and quinolinic acid) was found in 11 studies and a decrease in 3 studies. In studies on general glia cell density, 7 of 34 studies showed a decrease in glial cell density and 2 studies an increase. Furthermore, changes, mostly increases, in mRNA expression levels of chemokines and cytokines (interleukins, TNF-*α*, TNF-*α* receptor, and IFN-*γ*) were frequently found in postmortem studies of schizophrenic brains. Other proinflammatory markers such as substance P, NF-*κ*B, SERPINA3, and interferon-induced transmembrane protein (IFITM) were found elevated in concentration and/or expression in certain brain regions in schizophrenia patients in some, though not all, studies [32]. A similar meta-analysis of the literature on postmortems in mood disorders was somewhat more conclusive: in addition to findings of changes in the cytokine profile in the brain, it is argued that microglia and perivascular macrophages are found at increased density in the postmortem tissue. Meanwhile, oligodendroglia and GABAergic interneurons are found at a diminished number. One hypothesis that could explain this observation is that activated microglia release glutamate, which might damage neurons. The loss of oligodendroglia might also be a direct consequence of inflammation. So, pathways of neuroexcitation may be highly changed through neuroinflammatory mechanisms [33].

(III) In multiple studies elevations of CRP and/or proinflammatory cytokine levels were shown to return to normal when the psychiatric disorder was treated. For example, the level of IL-1*β* and IL-2 in depression patients returned to a similar level to that seen in controls after treatment with serotonin reuptake inhibitors over a 20-week period [34]. Accordingly, bipolar disorder patients treated with mood-stabilizers and antipsychotics responded with a significant decrease in high sensitivity CRP in the aforementioned study [14]. IL-1RA and IL-10 have also been found to be increased in drug-naïve schizophrenia patients decreased after 6 weeks of treatment with atypical antipsychotics. A strong correlation between IL-10 level and a score of negative, general, and total symptoms was found. Plasma levels of IL-2 and IL-6 were found to be decreased after antipsychotic treatment of schizophrenic patients in a meta-analysis of 8 similar studies [35]. Though little is said in the above studies on the correlation of patient outcome and the effects of pharmaceutics on cytokine levels, psychotherapy alone showed a decrease of IL-1Ra, IL-5,-6,-8,-10, G-CSF, IFN-*γ*, and TNF-*α* levels parallel to improvement of symptoms in major depression patients [36].

(IV) There is increasing evidence that some primary genetic risk factors for psychiatric disorders impact on immune functions. Most common psychiatric disorders are highly polygenic. Genome-wide association analysis of 5 common psychiatric disorders including schizophrenia, bipolar disorder, depression, ADHD, and autism identified genes in immune pathways as significantly contributing to risk [37]. The strongest and consistent association of SNPs for immune response and synaptic plasticity is associated with HLA region [38]. Recently, the most significant GWAS hit for schizophrenia risk (rs13194053), a SNP in the major histocompatibility complex region [39], was shown to exert risk in part through impacting on the gene encoding complement 4 (C4) [40]. Some rare but recurrent copy-number variants (CNVs) associated with mental illness also impact on immune functions, including the 22q11.2 deletion associated with velo-cardio facial syndrome, a topic we will return to below.

(V) Conversely, patients with CNS inflammation caused by infection or brain injury can develop symptoms similar to those seen in psychiatric patients. In a study of 83 patients with viral encephalitis 67% of them were presented with psychomotor agitation, 55% with drowsiness, 47% with disorientation, and 43% with visual hallucinations and 34% showed aggressiveness as a symptom. One year after hospital treatment in 70 of the same patients, 16% had memory disorders, 9% showed signs of aggressiveness, and 8% suffered from aphasia, 8% from visual hallucinations, and 7% from auditory hallucinations [41]. Brucellosis, typhoid fever, Lyme disease, Leptospirosis, and Whipple's disease are all examples of inflammatory diseases caused by infection that can be accompanied by psychiatric symptoms ranging from depression, mania, personality trait, and behavioral changes to manifest psychosis [42]. Regarding inflammation during healing after trauma, a survey of 722 outpatients after brain injury with an average of 10 days' unconsciousness after injury found a rate of DSM-IV valuable diagnosis of depression in 42% of patients [43]. While it is not possible to fully disentangle the causal direction in these associations, they are consistent with the view that infection and brain injury may lead to psychiatric symptoms via the triggering of inflammatory processes.

(VI) Increasing number of studies support the hypothesis that autoantibodies that bind mainly to neuronal membrane protein or synaptic proteins result in psychiatric disorders. To date, the clear causative role of autoantibodies on psychotic symptoms has been most clearly shown for the N-methyl-D-aspartate receptor (NMDA-R), although further studies are still needed [44–46]. Similarly autoantibodies against dopamine-2 receptor, which regulates movement and behavior, have been associated with paediatric autoimmune neuropsychiatric disorders associated with streptococcal infection and subjects with Tourette's syndrome [47]. Autoantibodies against antiopioid receptor, 5-hydroxytryptamine receptor 1A, and muscarinic cholinergic receptor 1 are thought to induce a depressive state in psychiatric illness [48, 49].

(VII) Activation of inflammation in animal models leads to behavioral abnormalities in a range of experiments. In rodent models, peripheral injection of inflammation-provoking lipopolysaccharide (LPS, 0.83 mg/kg) has been shown to generate sickness/depression-like symptoms, that is, prolonged period of immobility in forced swim test and tail suspension test. This effect was ascribed to an activation of the tryptophan-degrading enzyme indoleamine 2,3-dioxygenase [50]. Injecting animals with proinflammatory cytokines like IL-1a or TNF-*α* has also been shown to lead to sickness behavior in a dose- and time-related manner [51]. LPS injection to pregnant rats could be demonstrated to lead to pronounced changes of the offspring's brain development: cortical and hippocampus thickness were found increased and markers for neural and glia progenitors decreased and abnormally distributed over the brain regions. This was accompanied by an inflammation reaction within the brain, as indicated by the elevated presence of cytokines [52]. It has been suggested that microglia activation through LPS-stimulation may lead to a damage of oligodendrocyte progenitor cells in a way that ultimately leads to severe losses of oligodendrocytes and myelination during development [53]. It is remarkable in this context that immune system stimulating drugs like *β*-interferon have been found to induce psychiatric symptoms, for instance, in hepatitis C treatment [54, 55].

All the above points support the hypothesis that inflammation plays a significant role in the pathophysiology of major psychiatric disorders. However it remains unclear to what extent immune changes are primary in the causation of these conditions and to what extent they are a consequence of brain pathology.

Finding pathways that can initiate neuroinflammation may help us find mechanistic key links between prolonged stress exposure or traumatic life events in relation to the development of a psychiatric disease. For instance, overexcitation of neurons in epilepsy has been demonstrated to activate microglia and inflammation [56]. If the same observation holds true for situations of sensory overload, experienced as stress and emotionally overwhelming feelings, remains to be elucidated. Similarly, it is unclear if metabolic changes, like changes in circulation or hormone levels and receptors in straining situations, lead to oxidative stress in the brain, which would in turn provoke neuroinflammation.

The identification of the molecular mechanisms that connect inflammation and altered behavior will be important in the development of new treatments for neuropsychiatric disorders. Some emerging studies hypothesize that synaptic damage seen in psychiatric disorders may in part be a consequence of neuroinflammation, reflecting the situation seen in other conditions such as multiple sclerosis. Importantly, dysfunctional synaptic activity is associated with high activity of glutamatergic synapses and loss of GABAergic synapse function in schizophrenia and autism spectrum disorders. This imbalance seems to be provoked through cytokines that are upregulated during inflammation, very much like the ones we see in psychiatric disorders, supposedly released by activated microglia [57]. In most cases, inflammation within the CNS can activate an array of cellular and molecular changes including activation of astrocytes, oligodendrocyte dysfunction, and mitochondrial damage, changes in neurogenesis and neural circuits, and a general imbalance of neurotransmitters with a surplus of dopaminergic and a loss of glutamatergic neurotransmission, and it is also crucial for synaptic plasticity and pruning. Arguably, in concert these alterations can then lead to an impairment of behavioral functioning like cognition or emotion regulation [58].

## 3. Microglial Pathology in Psychiatric Disorders

At a cellular level, microglia and astrocytes regulate the central nervous immune response. During the early embryonic brain development, microglia are the first glial cells to develop alongside neurons [59] and they constitute *≅*10% of total glial cells in the adult brain [60]. Besides contributing to neuronal function [61, 62] microglia regulate the CNS response to antigens and inflammation [63], accompanied by cytokine and chemokine expression [64–67]. Following damage or stress, microglia located in intact areas elicit an immune response on the affected area by extending their processes. Microglial processes are highly dynamic and can sense the released ATP secreted from astrocytes and neurons in the damaged area [68]. Following activation microglia secrete two proinflammatory cytokines (IL-1*β* and TNF*α*), chemokines (CCL3, CCL5), and reactive oxygen/nitrogen species (nitric oxide, peroxides, superoxide, and peroxynitrite) (Figure 1) [69–71]. In turn, this neuroinflammation may cause damage to neurons and other glial cells. Aging is another key factor that influences the status of microglial state and the associated inflammatory condition. The gene expression profile in the aged brain indicates a clear upregulation of immune-related genes, including enhanced expression of IL-1*β*, TNF*α*, and IL-6 [72], suggesting that age related changes also lead to enhanced activation of microglial cells and supposedly inflammation, in the absence of external damage.

Besides being the first line of defense in the mammalian brain, microglia are important for synaptic formation and synaptic pruning [62, 73]. Microglia derived IL-10 has been shown to promote synapse formation. Reduction in the level of microglia during the postnatal stage or young adult stage of development results in reduced spine formation in the motor cortex. This reduction in synapse formation is associated with reduced motor performance and reduced levels of synaptic proteins like GluN2B and vGlut1 [74]. These studies suggest that microglia are important for learning and memory by promoting learning associated synapse formation [75]. A recent study has demonstrated early life infection can lead to an increase in microglial derived IL-1*β* level, which in turn modulates hippocampus-dependent learning and memory [76]. This observation is further supported by animal studies, where neonatal bacterial infection of rats induces hippocampus-dependent memory deficits in adulthood [77]. Microglia also eliminate nonfunctional synapses through the fractalkine receptor (CX3CR1) in the hippocampus [78, 79]. In retinal ganglion cells they regulate pruning through the C3 complement pathway [80]. In a recent study it was noted that increased expression of the C4 gene, encoding complement 4, was associated with high risk of schizophrenia. In C4-deficient mice, C4 promotes C3 activation which targets subsets of synapse elimination by microglia, suggesting its involvement in synaptic pruning [40].

Furthermore, TGF-beta signaling has been demonstrated to regulate microglial mediated pruning [81]. Microglia also modulate the sleep-wake cycle through extraction and retraction of the process [82]. It has been hypothesized that the microglial circadian clock might play a causative role in cognitive deficits and depression [83]. Exposure to environmental factors, especially during development, has been demonstrated to have profound effects on microglial state, inflammatory status of the brain, and cognition [84–86]. Although the underlying mechanism behind this in the context of neuropsychiatric disorders is poorly understood, fractalkine signaling at least in part seems to mediate microglial response to environmental risk factors [87].

Given the important role that microglia play in normal brain development and neuronal homeostasis, it is not surprising their dysfunction leads to neuropsychiatric disorders. Recent postmortem studies and PET imaging of peripheral benzodiazepine receptors provide evidence for microglial activation in the brains of patients with neuropsychiatric disorders such as schizophrenia, depression, and autism [88–95]. In schizophrenia, a significant increase in microglial density along with signs of activation in prefrontal and visual cortex has been reported [96, 97]. These results are highly promising, even though more specific markers of microglial activation are still needed to confirm these findings. One possible candidate for that might be translocator protein 18 kDa (TSPO), as decreased radiotracing was associated with schizophrenia-like behavior and inflammatory cytokine elevation in a mouse model [98]. Furthermore, in animal models of Rett syndrome (RTT), a devastating neurodevelopmental disorder, which is caused by a mutation in the X-linked* MECP2* gene encoding methyl-CpG-binding protein 2, microglia release an abnormally high level of glutamate, causing excitotoxicity that may contribute to dendritic and synaptic abnormalities in RTT [99]. Furthermore, in* Mecp2*-null mice a reduced number of microglia, which fail to phagocytose debris as effectively as those in wild-type mice, were found. These finding suggest that microglia may also be responsible for the disorder [100]. Stress-induced depressive-like condition in rodents has shown to be caused by reduced levels of microglia within the hippocampus and reduced expression of activation markers, which in turn might contribute to reduced hippocampal neurogenesis and increased depressive-like behavior [101]. Microglia expressing glucocorticoid receptors have also been shown to undergo extreme alterations when exposed to prolonged stress, like cell body shrinkage and debris accumulation in lysosomes [102].

Sustained microglial activation has been shown to contribute to the alleviation of symptoms associated with neurological disorders including psychiatric disorders (Figure 1) [103]. Enhanced proinflammatory cytokine response in concert with microglia activation occurs in response to external immune challenges and has been associated with cognitive and behavioral deficits in rodents [104–106]. As described earlier, such associations between proinflammatory cytokine levels in blood and psychiatric disorders are numerous in humans. It is feasible that microglia, the most important proinflammatory secreting cells in the brain, might be responsible for or at least involved in this increase in blood cytokine levels. It is interesting in this context that microglia also are very important postnatal development defining cells [107]. It is plausible to speculate that microglial state and functionality in the context of inflammation may be altered in patients with a genetic susceptibility to psychiatric disorder tighter with environmental factors? With induced pluripotent stem cell based disease modeling, one way to further investigate this question would be to derive microglia with a known genetic high-risk profile for psychiatric disorders, which will help to understand the mechanisms and triggers involved behind pathological inflammation. The potential mechanism of microglial activation that occurs during the course of psychiatric disorders is illustrated in Figure 1.

## 4. High-Risk Copy-Number Variants (CNVs): A Link between Genetic Alteration, Psychiatric Disorders, and Inflammation?

A strong association has been found between specific rare but recurrent chromosomal CNVs and psychiatric disorders, including schizophrenia, autism spectrum disorder, and mood and anxiety disorders [108]. Microarray experiments have now revealed abundant copy-number variations, a type of genetic variations in which stretches of DNA are duplicated or deleted [109, 110]. These structural alterations can range from 1 kilobase to several megabases and include either a single gene or contiguous sets of genes. CNVs can control the underlying psychiatric phenotype in multiple ways. They can affect the gene expression level by gene dosage effects or act as a faulty regulatory element to gene transcription cascades affecting other gene loci. They can also lead to frame shifts resulting in an abnormal genetic fusion product [111]. The mechanisms by which CNVs can lead to neuropsychiatric disorders and underlying complex behavioral traits are perhaps most clearly shown by rare and highly penetrant CNVs, involving the loss, gain, or disruption of a dosage-sensitive gene(s).

Some of the most common CNVs associated with neuropsychiatric disorder include chromosomal regions 1q21.1, 3q29, 15q11.2, 15q13.3, 16p11.2, 16p13.1, and 22q11 [112]. The locus 1q21.1 is associated with duplications in autism and with deletions or duplications in mental retardation [113–115]. The 15q13.3 locus is associated with deletions in mental retardation with seizures and deletions or duplications in autism and other neuropsychiatric disorders [116–118]. The most common microdeletion syndrome in humans, 22q11.2, results in an increased rate of a range of psychiatric disorders in children with other developmental and intellectual disabilities [119–122]. A 15q13.3 microdeletion in humans results in a range of neurodevelopmental/psychiatric disorders, including autism spectrum disorder, schizophrenia, epilepsy, and intellectual disability [116–118, 123–131]. Microduplication or deletion at the 16p11.2 loci is associated with schizophrenia, bipolar disorder, mental retardation, autism, seizure disorders, and psychosis in Alzheimer's disease [132–135]. De novo CNV analysis has resulted in enrichment of genes that are associated with the NMDAR network, GABAA receptor, abnormal CNS synaptic transmission, abnormal learning/memory/conditioning, and abnormal cued conditioning behavior [112, 136–139]. However, these studies have not picked up genes that can influence inflammatory condition underlying psychiatric symptoms.

There is strong evidence that deletions at 22q11.2 directly impact immune function. Thymic hypoplasia is a common feature associated with 22q11.2 deletions resulting in CD4^+^CD45RA^+^ T cell counts, which can result in immunodeficiency [140]. A recent study looking at a wide range of cytokines in patients with 22q11.2 deletions demonstrates a significant increase in the serum levels of proinflammatory and angiostatic chemokine IP-10 in patients with 22q11.2 deletions compared to healthy individuals. The increased IP-10 profile was associated with conotruncal congenital heart defects [141]. It is less certain how these immune changes might relate to the psychiatric symptoms seen in the disorder, but a positive association between severity of autism-related behaviors and level of serum concentrations of inflammatory cytokines IL-1*β*, interferon gamma, and IL-12p70 in individuals with 22q11.2 deletions has been reported [142]. Furthermore, toddlers with 22q11.2 deletion have an elevated level of CD3+, CD4+, and IL-4-IFN-*γ*+ lymphocytes as compared to healthy control, suggesting that 22q11.2 deletion is associated with dysregulated Th1 cytokine production such as IL-4 and IFN-*γ* [143]. However, if the dysregulated Th1 cytokine production is also present in adulthood needs to be investigated. A recent study looking at cellular modeling of neurodevelopment by differentiating of hiPSCs carrying 22q11.2 deletions into neuronal lineage reports a reduction in the level of neural differentiation propensity and neurite outgrowth and migration as compared to controls [144]. This phenotype was further associated with reduced expression level of miR-17/92 cluster and miR-106a/b cluster. A number of studies have established direct linkage of these clusters with dysregulated inflammation [145–147], suggesting that the underlying psychiatric symptoms associated with 22q11.2 deletions is at least in part associated with altered inflammatory state and the subjects are prone to an increased risk for a variety of autoimmune diseases [148].

TNF-*α* was found to be downregulated in expression in cells carrying the 15q13.3 microdeletion. TNF-*α* is known as a potent activator of inflammation [149]. It is plausible to speculate that genes that are involved in the 15q13.3 duplication are capable of altering TNF-*α* expression leading to an altered inflammatory state of the central nervous system. The CHRNA7 gene is one of the genes in 15q13.3 deletions. It encodes the nicotinic acetylcholine receptor alpha 7 subunit (*α*7nAChR), which is associated with schizophrenia in clinical studies and rodent models. A recent study using immortalized lymphoblastoid cell lines with 15q13.3 homozygous deletion has shown the CHRNA7 gene to modulate the production of TNF*α*. Furthermore, they identified STAT3 as one of the four genes that are differentially expressed following 15q13.3 deletions [149]. It has been demonstrated that STAT4 null mice showed impaired IL12-mediated functions [150]. IL-2 is an activator of the nitric oxide synthase [89], which has been demonstrated to be important for synaptic plasticity [151] suggesting that 15q13.3 deletion might modulate the synaptic plasticity through the STAT4/IL12/NOS pathway.

## 5. Modulating Neuroinflammation to Treat Psychiatric Disease

### 5.1. Theories on Pharmacological Effects of Anti-Inflammatory Drugs in Psychiatric Disorder

A number of drugs with anti-inflammatory features have been investigated either retrospectively or prospectively in terms of potential impacts on psychiatric symptoms including nonsteroidal anti-inflammatory drugs (NSAIDs) and the tetracycline related antibiotic minocycline. Aspirin, celecoxib, and the NSAID group of anti-inflammatory drugs, encompassing commonly prescribed drugs such as diclofenac, ibuprofen, or indomethacin, work by blocking the immune cascade enzyme cyclooxygenase (COX) [118], which result in reduced inflammation. COX is responsible for the translation of arachidonic acid into prostaglandins. COX-1, which is antagonized by classical NSAIDs, including diclofenac, ibuprofen and aspirin, is expressed ubiquitously in the body in addition to its role in inflammation. Celecoxib is a selective COX-2 inhibitor. COX-2 is mainly expressed during inflammation, but also in the brain and kidney [152]. In a rat model of neuroinflammation, created by injection of LPS, treatment with celecoxib significantly decreased the rate of microglia and astrocyte coactivation together with levels of IL-1*β*. Dopaminergic neurons and astrocytes with an upregulation of COX-2 were found in diminished numbers in treated individuals as compared to controls [153]. Inhibition of COX-2 has also been shown to lead to decreased production of kynurenic acid, which is a glycine antagonist at the NMDA-receptor and a nicotinic receptor antagonist. It has been found in elevated concentrations in schizophrenia patients' cerebrospinal fluid [154–156]. For minocycline, multiple effects seem to make up its anti-inflammatory character. In models of neuroinflammation, minocycline has been shown to inhibit activation and proliferation of microglia, as well as antagonizing important proinflammatory enzymes like COX-2, iNOS, NAPDH-oxidase, and P38 MAPK. It also seems to reduce T-leukocyte migration to the CNS by inactivation of MMP-9 (the enzyme rendering the brain-blood barrier permissive during inflammation).

**Figure 2:**
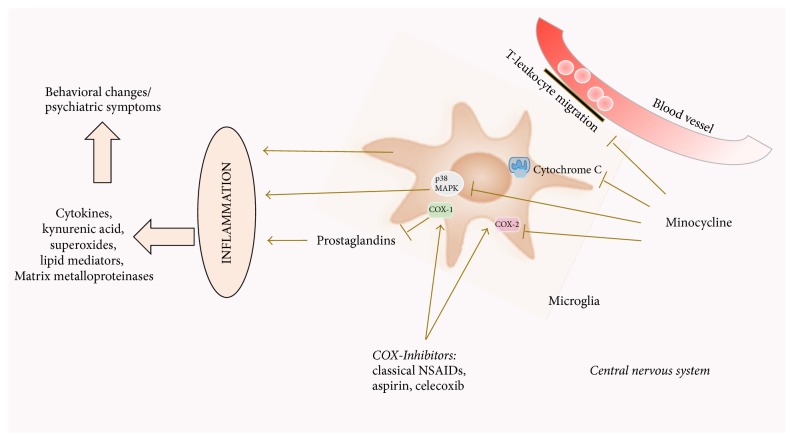
*Pictorial diagram summarizing the effects of anti-inflammatory medication on neuroinflammation mediated by microglia.* Inflammation, amplified through the activation of microglia, leads to the release of cytokines, kynurenic acid, superoxides, lipid mediators, and matrix metalloproteinases. These molecules may contribute to the pathological changes seen in neuropsychiatric conditions. Anti-inflammatory drugs like cyclooxygenase inhibitors block the prostaglandin-mediated cascade of this inflammation. Minocycline acts via (i) inhibition of T-leucocyte migration over the blood-brain barrier, (ii) cytochrome c inhibition in mitochondria, (iii) antagonism of MAPK p38 and subsequent products, and (iv) inhibition of COX-2 (COX: cyclooxygenase, MAPK: mitogen activated protein kinase).

In models of neurodegenerative disease, survival of neurons and glia cells can be increased through minocycline, through caspase dependent and independent mechanisms [157] (Figure 2). Little is known on why these drugs also work in psychiatric disorder, but one might speculate that the same cascades and factors of inflammation, notably microglia, are affected. We have summarized the clinical trials of compounds that aim to reduce the proinflammatory mediators in psychiatric disorders (Table 2).

### 5.2. Schizophrenia

In schizophrenic patients, several clinical studies exist on adjunctive treatment with drugs with anti-inflammatory features.

When 70 patients with at least a moderate symptom burden and a DSM-IV diagnosis for schizophrenia for under 10 years were treated with aspirin or placebo in addition to antipsychotic treatment, measurements by Positive and Negative Symptom Score (PANSS) showed an improvement in overall psychopathology and in positive symptoms. However, no differences in negative symptom or general scores were found [158]. In a similar study add-on aspirin given to patients improved symptoms, however only in patients with high CRP serum levels [159]. In patients with an acute exacerbation of schizophrenia treatment with cyclooxygenase-2 inhibitor, celecoxib in addition to risperidone led to a significant improvement of positive and negative symptoms in excess of the relief assignable to the antipsychotic [154, 161–163]. On the other hand, when patients who had suffered from symptoms for at least three months despite treatment, rather than patients with an acute exacerbation of psychosis, were treated with celecoxib in a similar study design, no difference in outcome was found [164]. So it might be that the effect of celecoxib add-on treatment in schizophrenia patients is dependent on the duration of the disorder. Looking at anti-inflammatory drugs as a possible risk or protective factor for psychiatric disorder, in a retrospective investigation looking at more than 2000 schizophrenic patients who had concomitantly taken nonsteroidal antirheumatic drugs or paracetamol, researchers found a higher risk for relapse to active psychosis. It is not clear whether this may be simply assignable to the parallel comorbidity treated with nonsteroidal anti-inflammatory drugs (NSAIDs) or the drug use per se [165]. Then, again, previous NSAID use was examined in a case-control trial (82 cases, 359 controls) of antipsychotics prescription. This showed a significant reduction of risk to develop psychosis when NSAID had been taken, but only in male individuals [166]. A nested case-control study of 1443 case events indicating schizophrenia exacerbation in patients using antipsychotics looked back at recent use of COX-2 inhibitors. The risk for schizophrenia exacerbation was not increased when the anti-inflammatory drug had been taken beforehand (for 0–93 days) [167].

Double-blind, randomized placebo-controlled studies showed that adjunctive treatment with the tetracycline minocycline to usual treatment could significantly improve negative symptoms [156, 168, 170]. Though unchanged by the treatment with minocycline, positive symptom score seems to be predictor for that improvement of negative symptoms [171]. Furthermore, minocycline seems to have positive effects on cognitive executive functions, that is, working memory, cognitive shifting, and planning [172]. A proportion of schizophrenic patient respond to clozapine. In a recent study positive outcome along with improved cognitive outcome has been reported when patient received minocycline together with clozapine in a 10-week double-blind, placebo-controlled trial involving 52 patients [193]. So minocycline add-on therapy seems especially interesting in negative and cognitive symptom treatment of schizophrenia.

Although the above data are interesting, it can only reflect implication of inflammation in pathogenesis of schizophrenia. Therefore, randomized prospective trials with a larger sample size and longer follow-up duration are crucial to establish anti-inflammatory therapy as an effective, safe, and tolerable add-on treatment in schizophrenia.

### 5.3. Bipolar Disorder

In bipolar disorder, investigations of proinflammatory cytokines, namely, TNF-*α*, INF-*γ*, IL-6, and high sensitivity CRP, found elevated levels in a cohort of 30 patients in acute mania as compared to healthy controls. Most significantly, only levels of CRP were decreased after treatment with antipsychotic drugs (± electroconvulsive therapy) while patient showed good outcomes [14]. In a study originally aiming at the examination of a potential drug blocking effect of NSAID on treatment with mood-stabilizers no difference in outcome for bipolar patients was found with and without NSAID [173].

A trial on the effect of aspirin on lithium-using patients with erectile dysfunction found no differences in depressive or mania symptoms before and with the add-on treatment [174]. Nevertheless, aspirin had beneficial effects in a pharmacoepidemiological studies looking at mood-stabilizer substance change or dosage increase in patients on lithium (interpreted as worsening of bipolar disorder) in correlation to the use of further drugs. Prescribed at a low-dose of 30 or 80 mg per day and for an unspecified time or for more than 1, 45, 90, or 180 days, the patients, picked from the nationwide Netherlands PHARMO Record Linkage System, had significantly fewer events of mood-stabilizer treatment alteration [175].

In 2008 Nery et al. investigated the effect of celecoxib treatment in 28 patients with a DSM-IV diagnosis of bipolar disorder during a depressive or mixed episode of the disease. The study was randomized, double-blind, and placebo-controlled and celecoxib was given out at a dosage of 400 mg per day over 6 weeks. This was in addition to a stable treatment with mood-stabilizers or atypical antipsychotics. Among the patients who finished the complete trial, a significant improvement of depressive symptoms (as measured by Hamilton Depression Rating Scale) could be found in the first week of celecoxib treatment [176]. Similarly, a study with celecoxib or placebo in 46 patients, in addition to sodium valproate treatment, showed that remission rates were significantly higher with the adjunctive treatment than without assessed by the young mania rating scale (YMRS) following treatment of 6 weeks. At day 42, YMRS scores were significantly lower in the celecoxib group [177]. Trials over multiple centres for treatment of psychiatric disease with add-on minocycline and/or celecoxib/aspirin are currently ongoing [194, 195].

The above adjunctive anti-inflammatory therapy has more efficacy and comparable tolerability compared with control and future prospective studies with a longer study duration which are based on larger sample sizes are needed to comprehensively evaluate the efficacy and tolerability of anti-inflammatory therapy in bipolar disorder.

### 5.4. Major Depression Disorder

Treatment of major depression with NSAID has been tried reluctantly so far because of reports in which parallel NSAID or COX-2 inhibitor treatment of comorbidity lead to supposed drug interaction and higher resistance level to antidepressant treatment [196]. However, there is a number of noteworthy, even if sometimes contradictory, clinical trials that frequently come from investigations of anti-inflammatory comorbidity treatment. In a study on the effect of NSAID use in addition to escitalopram or nortriptyline treatment in a cohort of 811 major depression patients no significant effect of NSAID use on the outcome of the antidepressant therapy was found [178]. An Australian study in over 5000 men of older age (69–87 years) investigated the correlation between aspirin use and development of depressive symptoms. Individuals who had used aspirin as antiplatelet agent drug in the past but had stopped the treatment before the mood assessment were at a significantly higher risk for higher scores of depression [179]. However, a population-based study of 1631 patients could not find any lower rates of depression in patients who had taken aspirin on a regular basis [180].

A meta-analysis of 4 randomized controlled trials with celecoxib adjunctive to treatment with antidepressants was able to show consistent improvements in depression scores and remission and response rate [181]. Similarly, in 52 outpatients with breast cancer and concomitant depression a trial compared outcome after treatment with diclofenac versus celecoxib. At the endpoint, both treatments showed significant improvement as indicated by HDSR score. The celecoxib treatment was significantly more successful than the diclofenac treatment, while the analgesic effect was comparable [182]. Patients with depression due to Brucellosis showed significant response to treatment with celecoxib (200 mg bid) as measured by Hamilton Depression Rating Scale in a study on 40 patients over 8 weeks [183].

Then, again, in the Alzheimer's Disease Anti-inflammatory Prevention Trial, a study in more than 2000 individuals older than 70 years and with a family history of Alzheimer's disease (though without any cognitive dysfunction), the application of celecoxib or sodium naproxen was tested against placebo. In the beginning of the trial, around 20% of the test persons had an increased score for depression as measured by 30-item Geriatric Depression Scale. However, there was no significant change in that percentage over the trial, or in scores of individual probands [184]. Thus, one could argue that the effect of celecoxib might be age-dependent.

A 6-week open-label study in 25 inpatients with a DSM-IV diagnosis of major depression with psychotic features showed that minocycline (150 mg/day) as adjunctive therapy to fluvoxamine, paroxetine, or sertraline was beneficial. Both depressive and psychotic symptoms were significantly improved [185]. Similarly, HIV positive patients (*n* = 46) with mild-to-moderate scores of depression (Hamilton Depression Rating Scale) were treated with 100 mg bid minocycline or placebo in a double-blind randomized trial. A significant reduction of depression score was observed by the end of six weeks [186].

### 5.5. Anxiety/Anxiety Disorder

Even though to our knowledge there are no clinical trials with celecoxib, other NSAIDs, or tetracyclines in anxiety disorder patients to date, there are some interesting observations on anti-inflammatory manipulation of the immune system in animal models of anxiety symptoms. One trial in experimental autoimmune encephalitis in a rat model of multiple sclerosis showed that gene treatment with IL-10 diminished anxiety and depression symptoms, indicated by voluntary wheel running frequency [197]. In mouse models of anxiety after traumatic brain injury [198] and of anxiety following neonatal immune activation by infection treatment with minocycline led to a significantly positive effect on psychopathologic outcome [199].

A recent study provides evidence of high levels of IFN-*γ* and TNF-*α* but low IL-10 serum levels in anxiety patients when compared to healthy control subjects [200]. Furthermore, a large cohort study looking at the association between anxiety disorders and inflammation identified an elevated level of CRP in male patients with anxiety disorders [201]. In another study which included patients with rheumatoid arthritis receiving anti-TNF-*α* therapy had less frequent prevalence of any mood or anxiety disorders [202] demonstrating a beneficial role of anti-inflammatory therapy in anxiety disorders. Coumarin-derivate esculetin [203] as well as phenolic-rich* Aronia melanocarpa* berry juice attenuated anxiety and depressive symptoms in mice and rats, as measured during LPS-inducement and stress test, respectively. Both substances are known for their antioxidative power and it is thus feasible that the effect is assignable to an anti-inflammatory impact [204].

A synthetic derivative (4-phenylselenyl-7-chloroquinoline) with anti-inflammatory and antioxidative potential was shown to have anxiolytic effects in mice, as measured by elevated-plus maze and light dark tests. The quinolone was applied 0.5 hours prior to onset and anxiety-related behavior seemed avoided for up to 72 h. However, the authors of this study assigned the effect primarily to a change in glutamate levels provoked by the drug rather than anti-inflammatory effect [205]. The polyphenol Honokiol is primarily known as a drug used in traditional Chinese medicine. In mice, application of Honokiol (for 2 days at either 2 or 5 mg/kg i.p.) prior to induction of inflammation by LPS injection led to a decrease of anxiety levels, as measured by behavioral testing using the elevated-plus maze and open field test. Furthermore, a significant decrease in plasma levels of IL-1*β*, IL-6, TNF-*α*, and BDNF was found in those mice pretreated with Honokiol as well as a liver-protective effect [206]. Of note, physical exertion is shown by several lines of evidence to have anti-inflammatory effects and at the same time is one of the most effective nonpharmaceutical remedies of anxiety [207].

### 5.6. Obsessive-Compulsive Disorder

In a double-blind randomized trial in 50 OCD outpatients, fluvoxamine in combination with anti-inflammatory COX-2 inhibitor celecoxib (200 mg bid) showed a significant improvement of symptoms in comparison with fluvoxamine alone and placebo control [187]. In a similarly designed study 48 OCD patients who were nonresponders to serotonin reuptake inhibitors were add-on treated with N-acetylcysteine (up to 2400 mg/d) or placebo over 12 weeks. At the end of this period 15% more than in the placebo group were responsive to therapy in the N-acetylcysteine group (52.6%) [188].

In an open-label study, 9 treatment-resistant outpatients with OCD were given 100 mg/day of minocycline adjunctive to their treatment with serotonin reuptake inhibitors. In diagnostic testing by Yale–Brown Obsessive-Compulsive Scale every 2 weeks over 12 weeks, no significant improvements in outcome could be found in the cohort as a whole. However, in 2 patients with early-onset OCD, more than 30% reduction of the Yale–Brown Obsessive-Compulsive Scale was reached, which was initially defined as a marker for treatment response [189]. Indeed, in a double-blind randomized trial on 102 OCD patients, therapy with minocycline (100 mg bid) as add-on to fluvoxamine (100 mg/day for the first two days and then 200 mg/day for the remaining 6 weeks of the trial) was associated with a significantly higher rate of patients responding completely or partially to the treatment. Measured by the Yale–Brown Obsessive-Compulsive Scale, total score and obsession subscale score were significantly lower in the minocycline than in the placebo group [190].

### 5.7. Autism Spectrum Disorder

Little is found on anti-inflammatory treatment in autism spectrum disorder so far. Add-on of celecoxib (up to 300 mg/day) to risperidone treatment in a study on 40 children suffering from autism leads to significant improvements of scores in irritability, social withdrawal, and stereotypy [191]. However, an open-label study in 11 children on treatment with 1.4 mg/kg body weight of minocycline found no clinical improvements after 6 months (measured using Clinical Global Impression Severity Scale, Clinical Global Impression Severity Scale Improvement, and Vineland Adaptive Behavior Scales). IL-8 was found significantly decreased in serum and cerebrospinal fluid, while other cytokines, that is, TNF-*α*, CD40L, IL-6, IFN-*γ*, and IL-1*β*, were unchanged [192].

## 6. Conclusions

A close examination of the literature demonstrates that the increased understanding of the microglia driven inflammatory effect on psychiatric diseases has not yet been translated into the clinical treatment for these disorders. The results from experimental animal models as well as from patients cohort studies are however quite promising. Adjunctive therapy with anti-inflammatory medication, especially in cases where conventional therapy has failed to bring complete recovery, may provide a novel route to treatment of these conditions.

## Figures and Tables

**Table 1:** Circulating cytokines and acute phase proteins that are found elevated in different psychiatric disorders.

Psychiatric disorder	Cytokines/acute phase proteins elevated
Schizophrenia	IL-1*β*, IL-6, IL12-p70, sIL-2R, TNF-*α*, IFN-*γ* [8, 9]
Depression	IL-1, IL-6, sIL-1R, sIL-2R, sIL-6, TNF-*α*, CRP [10, 11]
Bipolar disorder	IL-4, IL-6, IL-10, sIL-2R, sIL-6R, TNF-*α*, sTNFR1, IL-1 receptor antagonist, INF-*γ*, hs-CRP [13–15]
Anxiety disorder	IL-6, IL-8, IL-10, IL-18, MCP-1, MMP2, TNF-*β* [12]^*∗*^
OCD	IL-2, IL-4, IL-6, IL-10, TNF-*α*, sTNFR1, sTNFR2 [16, 17]
PTSD	IL-1*α*, IL-1*β*, IL-2, IL-4, IL-6, IL7, IL-8, IL-10 and, IL12p40, IL12p70, IL-13, IL-15, TNF-*α*, MIP-1*α*, GM-CSF, IP-10, eotaxin [18, 19]^*∗∗*^
Autism	IL-1*β*, IL-1RA, IL-5, IL-8, IL12p40, IL-12(p70), IL-13, IL-17, GRO-*α* [20, 21]

^*∗*^After stimulation with LPS; ^*∗∗*^in PTSD and panic disorder patients.

**Table 2:** Clinical studies on use of anti-inflammatory drugs in treatment of psychiatric disorder. *N*: proband number, *T*: time frame, PANSS: Positive and Negative Symptom Score, SANS: Schedule for the Assessment of Negative Symptoms, and CGI score: clinical global impression score.

Treatment	Psychiatric disorder	Probands	Trial design	Outcome	Authors
Aspirin 1000 mg/day plus 40 mg/day pantoprazole, in addition to antipsychotic treatment	Schizophrenia	Patients with at least moderate symptom burden and a DSM-IV diagnosis for schizophrenia spectrum disorder for under 10 years *N* = 70	Randomized controlled trial *T* = 3 months	Mixed model improvement in overall psychopathology and in positive symptoms as measured by PANSS No differences in negative symptom or general scores	Laan et al., 2010 [158]

Aspirin 1000 mg/day plus 40 mg/day pantoprazole, in addition to antipsychotic treatment	Schizophrenia	Patients with moderate or above on CGI score, moderate score on two of PANSS items: delusions, hallucinatory behaviors, conceptual disorganization or suspiciousness/persecution, and/or a total PANSS negative symptoms score above 18 *N* = 400	Post hoc analysis of randomized controlled trial *T* = 16 weeks	Improvements in patients with high CRP levels	Weiser et al., 2014 [159]

Celecoxib 200 mg bid in addition to 2–6 mg/day risperidone	Schizophrenia	Patients with acute exacerbation of schizophrenia *N* = 50	Double-blind, Randomized Placebo-controlled *T* = 5 weeks	Significant improvement of positive symptoms	Müller et al., 2002 [160]

Celecoxib in addition to olanzapine	Schizophrenia	Patients with acute exacerbation of schizophrenia *N* = 94	Open-label, prospective, controlled *T* = 6 weeks	Improvement of positive, negative, and general psychopathology and total scores as measured by PANSS	Baheti et al., 2013 [161]

Celecoxib (200 mg bid) in addition to risperidone (200 mg/day)	Schizophrenia	inpatients diagnosed with active phase schizophrenia *N* = 60	Double-blind randomized placebo-controlled *T* = 8 weeks	Significant improvement regarding positive symptoms as measured by PANSS	Akhondzadeh et al., 2007 [162]

Celecoxib	Schizophrenia	Patients with a first manifestation of schizophrenia *N* = 49	Double-blind randomized placebo-controlled *T* = 6 weeks	Significant improvement regarding positive and negative symptoms as measured by PANSS	Müller et al., 2010 [163]

Celecoxib (400 mg/day) in addition to antipsychotic treatment	Schizophrenia	Outpatients with a DSM-IV diagnosis of schizophrenia, experiencing persistent symptoms despite treatment for 3 months *N* = 38	Double-blind, randomized, placebo-controlled, *T* = 8 weeks	No difference in outcome, as measured by PANSS	Rapaport et al. 2005 [164]

Concomitant intake of NSAID or paracetamol	Schizophrenia	Schizophrenia patients *N* = 2000	Retrospective investigation	Higher risk for relapse to active psychosis	Köhler et al., 2016 [165]

Previous NSAID use	Schizophrenia	Patients prescribed antipsychotics 82 cases, 359 controls	Case-control trial of antipsychotics prescription	Significant reduction of risk to develop psychosis when NSAID had been taken, but only in male individuals	Laan et al., 2007 [166]

Use of COX-2 inhibitors	Schizophrenia	Case events indicating schizophrenia exacerbation in patients using antipsychotics *N* = 1443	Nested case-control study *T* = previous 93 days	No increase of risk for schizophrenia exacerbation	Stolk et al., 2007 [167]

Minocycline in addition to usual treatment	Schizophrenia	Patients with early-stage schizophrenia *N* = 94	Double-blind, randomized, placebo-controlled *T* = 1 year	Significant improvement of negative symptoms as measured by PANSS	Chaudhry et al., 2012 [168]

Minocycline (200 mg/day) in addition to risperidone	Schizophrenia	Patients with DSM-IV diagnosis of early-phase (less than 5 years) schizophrenia who had been on a steady dosage of risperidone *N* = 93	Double-blind, randomized, placebo-controlled *T* = 16 weeks	Significant improvement in treatment response in the Scale for the Assessment of Negative Symptoms (SANS) total scores and PANSS for negative symptoms	Liu et al., 2014 [169]

Minocycline (200 mg/day)	Schizophrenia	Patients with DSM-IV diagnosis of schizophrenia, no treatment with therapeutics for one week before start of trial *N* = 43	Randomized, placebo-controlled *T* = 8 weeks	Significant decrease of SANS score after 8 weeks (though not 4 weeks) of treatment	Ghanizadeh et al., 2014 [170]

Minocycline (up to 200 mg/day) in addition to risperidone (up to 6 mg/day)	Schizophrenia	Chronic schizophrenia patients *N* = 40	Double-blind, randomized, placebo-controlled *T* = 8 weeks	Significant improvement of negative symptom as measured by PANSS	Khodaie-Ardakani et al., 2014 [171]

Minocycline	Schizophrenia	Early-phase schizophrenia patients *N* = 54	Double-blind, randomized, placebo-controlled *T* = 6 months	Significant improvement of negative symptoms and general outcome measured by PANSS, Clinical Global Impression (CGI) and insight score, and improvements of cognitive executive functions	Levkovitz et al., 2009 [172]

NSAID and paracetamol	Bipolar disorder	DSM-IV-TR diagnosis of bipolar disorder I or II and at least mild symptoms *N* = 482	Secondary analysis from the Bipolar CHOICE study *T* = 6 months	No difference as measured by CGI-BP	Köhler-Forsberg et al., 2017 [173]

Aspirin	Bipolar disorder	Patients taking lithium with erectile dysfunction *N* = 32	Double-blind, randomized, placebo-controlled *T* = 6 weeks	No differences in mania or depressive symptoms, significant improvement of erectile dysfunction	Saroukhani et al., 2013 [174]

Aspirin, low-dose of 30 or 80 mg per day and for an unspecified time or for more than 1, 45, 90, or 180 days, in addition to lithium	Bipolar disorder	Patients with at least five previous prescriptions for lithium and at least 1-year drug history *N* = 5145	Pharmacoepidemiological study on the PHARMO Record Linkage System (RLS) in the Netherlands *T* = 10 year period	Significantly fewer events of mood-stabilizer treatment alteration	Stolk et al., 2010 [175]

Celecoxib (400 mg/day)	Bipolar disorder	DSM-IV diagnosis of bipolar disorder during a depressive or mixed episode of the disease *N* = 28	Double-blind, randomized, placebo-controlled *T* = 6 weeks	Improvement of depressive symptoms (as measured by Hamilton Depression Rating Scale) found in the first week	Nery et al., 2008 [176]

Celecoxib in addition to sodium valproate	Bipolar disorder	Patients with diagnosis of acute bipolar mania with manic episode without psychotic features *N* = 46	Double-blind, randomized, placebo-controlled *T* = 6 weeks	Significantly higher remission rates as measured by young mania rating scale	Arabzadeh et al., 2015 [177]

Concomitant NSAID use in addition to escitalopram or nortriptyline	Depression	Patients with major depression disorder treated in the GENDEP study *N* = 811	Retrospective on the GENDEP study *T* = up to 12 weeks	No change of serotonin reuptake inhibitor treatment	Uher et al., 2012 [178]

Previous aspirin use as anti-platelet treatment	Depression	Men of older age (69–87 years) *N* = 5273	Retrospective analysis of aspirin use after assessment of mood disorder symptoms *T* = last 5 years	Significantly higher risk for depression in individuals who had used aspirin as antiplatelet agent drug in the past, but had stopped the treatment before the mood assessment (measured by Geriatric Depression scale)	Almeida et al., 2010 [179]

Regular aspirin or statin use in the past	Depression	Depression patients *N* = 1631	Population-based study *T* = last 6 months	No higher risk for development of depression	Glaus et al., 2015 [180]

Celecoxib in addition to antidepressants	Depression	Patients with depressive episodes *N* = 150	Meta-analysis of 4 randomized controlled trials	Improvements in Hamilton Rating Scale for Depression scores, remission and response rate	Na et al., 2014 [181]

Diclofenac (50 mg bid) versus celecoxib (200 mg bid)	Depression	Outpatients with breast cancer and concomitant depression *N* = 52	*T* = 6 weeks	Significant improvements by HDSR score in both, celecoxib significantly more successful than diclofenac, analgesic effect comparable	Mohammadinejad et al., 2015 [182]

Celecoxib (200 mg bid)	Depression	Patients with depression due to brucellosis *N* = 40	Double-blind, randomized, placebo-controlled *T* = 8 weeks	Significant improvements on HDRS	Jafari et al., 2015 [183]

Celecoxib (200 mg bid) or sodium naproxen (220 mg bid)	Depression	Individuals older than 70 years and with a family history of Alzheimer's disease (though without any cognitive dysfunction) *N* = 2312	Alzheimer's Disease Anti-Inflammatory Prevention Trial Yearly follow-up	No significant change in individual or overall depression score measured by 30-item Geriatric Depression Scale	Fields et al., 2012 [184]

Minocycline (150 mg/day) as adjunctive therapy to fluvoxamine, paroxetine, or sertraline	Depression	Inpatients with a DSM-IV diagnosis of major depression with psychotic features *N* = 25	Open-label study *T* = 6 weeks	Significant improvement of depressive and psychotic symptoms as measured by Hamilton Depression Rating Scale and Brief Psychiatric Rating Scale	Miyaoka et al., 2012 [185]

100 mg bid minocycline	Depression	HIV positive patients with mild-to-moderate scores of depression *N* = 46	Double-blind, randomized, placebo-controlled *T* = 6 weeks	Significant reduction of depression score (Hamilton Depression Rating Scale)	Emadi-Kouchak et al., 2016 [186]

Celecoxib 200 mg bid in addition to fluvoxamine	OCD	OCD outpatient *N* = 50	Double-blind, randomized, placebo-controlled *T* = 10 weeks	Significant improvement of symptoms	Shalbafan et al., 2015 [187]

N-Acetylcysteine (up to 2400 mg/day)	OCD	OCD patients, nonresponsive to serotonin reuptake inhibitors *N* = 48	Double-blind, randomized, placebo-controlled *T* = 12 weeks	15% higher response rate	Afshar et al., 2012 [188]

Minocycline (100 mg/day) in addition to serotonin reuptake inhibitor	OCD	Treatment-resistant outpatients with OCD *N* = 9	Open-label study *T* = 12 weeks	No significant improvements in outcome in cohort as a whole, more than 30% reduction of the Yale–Brown Obsessive Compulsive Scale	Rodriguez et al., 2010 [189]

Minocycline (100 mg bid) as add-on to fluvoxamine (100 mg/day for the first two 200 mg/day for the remaining 6 weeks of the trial)	OCD	OCD patients *N* = 102	Double-blind, randomized, placebo-controlled *T* = 10 weeks	Significantly higher treatment response rate (either complete or partial) measured by the Yale-Brown Obsessive compulsive scale. Specifically patients scored significantly lower in total and on the obsessive subscale	Esalatmanesh et al., 2016 [190]

Celecoxib (up to 300 mg/day) in addition to risperidone	Autism	Children with autistic disorder *N* = 40	Double-blind, randomized, placebo-controlled *T* = 10 weeks	Significant improvements of scores in irritability, social withdrawal, and stereotypy	Asadabadi et al., 2013 [191]

Minocycline (1.4 mg/kg body weight)	Autism	Children with autism spectrum disorder *N* = 11	Open-label *T* = 6 months	No clinical improvements (Clinical Global Impression Severity Scale, Clinical Global Impression Severity Scale Improvement, and Vineland Adaptive Behavior Scales)	Pardo et al., 2013 [192]
